# Characterization and Structural Performance in Bending of CLT Panels Made from Small-Diameter Logs of Loblolly/Slash Pine

**DOI:** 10.3390/ma11122436

**Published:** 2018-11-30

**Authors:** Vanesa Baño, Daniel Godoy, Diego Figueredo, Abel Vega

**Affiliations:** 1 Instituto de Estructuras y Transporte (IET), Facultad de Ingeniería, Universidad de la República, Montevideo 11300, Uruguay; diego.figueredo.a@gmail.com; 2 Instituto de Ensayos de Materiales (IEM), Facultad de Ingeniería, Universidad de la República, Montevideo 11300, Uruguay; dgodoy@fing.edu.uy; 3 Forest and Wood Technology Research Center (CETEMAS), Asturias 33936, Spain; avega@cetemas.es

**Keywords:** cross-laminated timber, CLT, characterization, structural performance, small-diameter logs, thinning operations, *Pinus taeda/Pinus elliotii*, bending tests

## Abstract

The main objective of this work was to study the structural viability of using small-diameter logs of Uruguayan Loblolly/Slash pine, mainly from thinning operations, to design cross-laminated timber (CLT) panels. A visual grade named “CTH” (coniferous thinning) was proposed, and 45 specimens of sawn timber boards were tested, resulting in 51% lower bending strength than that of the minimum strength class C14. Subsequently, 20 CLT panels were manufactured and experimentally tested, the results showed that the bending strength of the CLT panels was 43% above that of the individual layers. Additionally, the structural performance of the CLT panels for use in floors was calculated, and the thickness-span relationship depending on strength class and imposed load are presented. Results showed than the use of CTH timber to design CLT floors implies a volume (m^3^/m^2^) 17% higher than that using C24 timber.

## 1. Introduction

Timber construction is an increasing global phenomenon as both the result of environmental policies and the need to increase construction productivity [1,2]. Such policies apply not only to the single-family home building sector, but also to multistory building sector too. In Finland, wood promotion campaigns have led to a 9% increase in the market share of timber multistory buildings between 2011 and 2015 [3]. More than 30 seven-floor mass-timber buildings have been built worldwide since 2008 [4], mainly using cross-laminated timber (CLT) [5]. Therefore, research is focused in the proposal of design procedures of tall buildings [5,6], design in seismic regions [7,8], and design of efficient connections between the panels [9,10]. CLT is an engineered wood product, developed in Europe in the 90s, which is manufactured from an odd number of orthogonal layers of sawn boards glued together with a structural adhesive under pressure, and is commonly used as flooring and walls in mass-timber buildings. The number of layers usually varies between three and nine, with each layer having a thicknesses of up to 40 mm. The mechanical properties of the sawn timber generally used in Europe (*Picea* spp.) for the longitudinal layers is classified as strength class C24 [11], although it may vary between C16 and C30 [12]. According to EN 338 [13], these strength classes correspond to a bending strength and longitudinal modulus of elasticity of 24 MPa and 11.0 GPa, respectively.

The production of this structural system requires large volumes of wood, so the raw material needs to be readily available and economical. Since CLT building is increasing worldwide [11], many authors have studied the viability of manufacturing CLT panels using local resources, mainly from fast-growing species with low mechanical properties. As such, there have been studies on the use of radiata pine in New Zealand [14] and in Chile [15], poplar (*Populus* spp.) in Hungary [16], and Southern pine [17] in the United States. The structural behavior of CLT panels made with UK Sitka spruce of strength class C16 [13] was studied [18], as well as CLT made with sugi (Cryptomeria japonica) from Japan [19]. This latter study made CLT using three different grades of sawn timber, whose modulus of elasticity varied between 3.5 and 8.0 GPa. In the same vein, fast-growing Uruguayan pine has been identified as a potential species for the manufacture of CLT panels. In addition to the characterization of CLT panels in bending for flooring use, many researchers have studied the in-plane performance of CLT as shear walls [20,21,22]. 

Forestry in Uruguay for commercial purposes began in the 90s, with plantations of both *Pinus* spp. and *Eucalyptus* spp., although no structural engineered wood products are as yet being commercialized. Two silvicultural systems have been described for Loblolly/Slash pine (*Pinus taeda/elliottii*), [23]: (i) 12-year rotation, principally destined for use as pulp and fiber or in particle board; and (ii) 24-year rotation for the production of sawlogs of higher commercial value. The 24-year rotation commonly includes two pruning operations (at 3 and 5 years after planting) and two commercial thinning operations (at 10 and 15 years). However, both juvenile and mature wood, from commercial thinning operations and clear cutting at the end of the forestry cycle, respectively, are commercialized as the same product by the sawmills. 

The current average annual production of Uruguayan pine wood is 2.9 million m^3^ [24]. Since 1.7 million m^3^ of that wood has no current industrial application and 1.4 million m^3^/year of that unused wood comes from silvicultural thinning [24], the use of thinning timber to produce CLT is a clear commercial opportunity. However, before this can happen, it is necessary to determine the strength and stiffness properties of CLT panels made from small-diameter logs of Uruguayan Loblolly/Slash pine, which come mainly from thinning operations. EN 16351 [25] specifies that two different approaches can be used to determine the strength and stiffness properties of CLT: (i) through determining the properties of the individual layers (sawn boards); and (ii) by testing of CLT panels themselves. However, unlike in the standard EN 14080 [26] for glued laminated timber (GLT), EN 16351 [25] does not provide the equations that are required to determine the strength and stiffness properties of CLT panels and, therefore, the strength classes of CLT panels are not defined. To this end, Unterwieser et al. [27] presented the characteristic values of strength and stiffness of CLT with the aim of facilitating the regulation of CLT panels by strength classes, and the next Eurocode updates will include the design of CLT and define strength classes for CLT elements [28]. 

In terms of structural calculations, the strength and stiffness properties of CLT are commonly estimated from analytical approaches that use the structural properties of the sawn timber boards that make up the layers, as mentioned above. Various methods are used to determine the moment of inertia of CLT panels and, thus, the corresponding modulus of elasticity. The most commonly used theories are the Timoshenko beam theory, the Shear Analogy Method, and the Mechanically Jointed Beams Theory (also known as the Gamma method); the latter being the most common method adopted in Europe [29]. Annex B of Eurocode 5 [30] develops the Gamma method for mechanically jointed beams. It is generally accepted that the Gamma method is applicable for the design of CLT members in bending [31], considering that only the longitudinal layers are load-carrying, and that the cross layers act as flexible shear connections. Shear analogy method [32] is more accurate, where both vertical shear deformations of the longitudinal layers and rolling shear deformations of the cross layers are considered. It requires the use of a plane frame analysis program, especially for the design of two-way spanning-floor elements or when dealing with concentrated loadings and special floor openings, or when dealing with CLT panels made of more than five layers. Therefore, the main difference between the last two methods is that the Gamma method considers the shear deformations of the cross layers only, while the Shear Analogy Method also takes into account the shear deformations of the longitudinal layers. 

The main objective of this work was to study the structural viability of using small-diameter logs of Uruguayan Loblolly/Slash pine mainly from thinning operations, which has low mechanical properties, to design CLT panels. To achieve this, it is necessary to characterize the sawn timber and the CLT panels and to study the relationship between the structural properties of the layers themselves and those of the CLT panel as a whole. Thus, a sample of 45 specimens of sawn timber boards and another of 20 specimens of 3-layer CLT panels were tested in bending, and the characteristic values for the individual samples and for the population were estimated according to [25,33,34]. Finally, the structural performance of the Uruguayan CLT was compared with that of the most common internationally commercialized CLT.

## 2. Materials and Methods

### 2.1. Materials and Visual Grading of the Sawn Timber

Sawn timber boards with a cross-section of 34 × 127 mm^2^ and from Uruguayan Loblolly/Slash pine plantations were selected in the sawmill. Boards came from trees with a diameter in thin tip greater than 200 mm, due to a limitation of the processing capacity of the sawmill, and from trees extracted mainly in commercial thinning. Since the sawmills do not make a commercial differentiation between trees coming from thinning (between 10 and 15 years) and those from clear cutting (at 24 years), a minor percentage of trees from the final cut were included in the sample. They were graded, following a previously published categorization system [35], with boards being designated as either: (i) “EC7”, which corresponds to strength class C14 [13], or (ii) “Reject” for structural purposes. Since knots are the main singularities that usually define the bending strength of softwood timber beams [35,36], these rejected boards were then re-graded according to a proposed new structural grade designated “CTH” (coniferous thinning), which permitted surface knots with a maximum diameter of up to 4/5 of the height of the board (*h*), and an edge knot diameter equal to the board’s width (*b*). The study sample of the present work comprised 11% of timber graded as EC7 and 89% as CTH.

### 2.2. Bending Tests of the Sawn Timber

**Figure 1:**
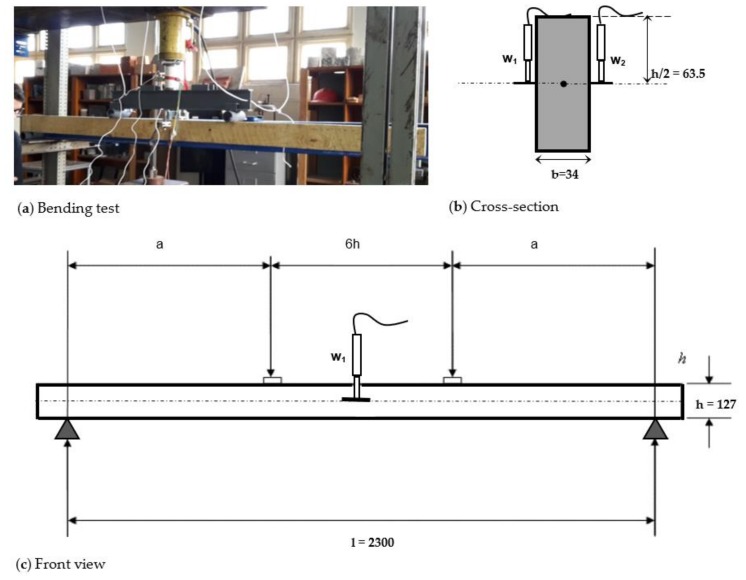
Four-point bending test of sawn boards: (**a**) bending test, (**b**) cross-section, and (**c**) front view (dimensions in mm).

Forty-five specimens of 2.4 m span (*l*) were tested in a four-point bending test following the recommendations of EN 408 [37] as shown in Figure 1. Global deformation was calculated as the mean value of the deflections measured in the neutral axis of both sides of the specimens by linear variable differential transformers (LVDT, DATA Instruments Inc., Acton, MA, US). The global modulus of elasticity can be determined from the global deflections measured in bending tests (Figure 1) by Equation (1) [37], when the value of shear modulus (*G*) is infinite. However, if the shear modulus is known, or if it is assumed as 650 MPa as proposed by EN 408 [37] for softwood, the local modulus of elasticity is deduced from Equation 1. A more accurate estimation of the local modulus of elasticity of Uruguayan pine can be derived from Equation (1) considering the ratio E/G = 16 proposed by EN 338 [13]. Bending strength was calculated for each specimen according to Equation (2) [37].

(1) $$E_{m}=\frac{3al^{2}-4a^{3}}{48I_{i\_ST}\left(\frac{w_{2}-w_{1}}{F_{2}-F_{1}}-\frac{3a^{3}}{5Gbh}\right)}$$

(2) $$f_{m}=\frac{3F_{a}}{bh^{2}}$$

where, *h* and *b* are the height and width of the specimen, respectively; *a* is the distance between a support and the closest load point; *I_i_ST_* is the net moment of inertia according to Steiner’s Theorem, which for a solid board of rectangular cross-section is equal to (*bh*^3^/12); *F*_1_ and *F*_2_ are the initial and final loads (kN), respectively, in the linear elastic phase; *w*_1_ and *w*_2_ are the mid-span deflections corresponding to *F*_1_ and *F*_2_, respectively; *G* is the shear modulus (*G* = *E_m_*/16); and *F* is the maximum load applied to the system at failure (each load point is *F*/2). 

### 2.3. Bending Tests of the Sawn Timber

The characteristic values of the sawn timber boards from thinning were obtained following the specifications of EN 384 [33] and EN 14358 [34]. Bending properties and density were analyzed for: (i) the individual specimens; (ii) the whole sample; and (iii) the population.

(i) Specimens. The modulus of elasticity of each specimen was adjusted to a reference moisture content (MC) of 12%, and the bending strength of each specimen (*f_m,i_*) was adjusted to a reference height (k_h_) of 150 mm. 

(ii) Sample. The 5th percentile of bending strength and density and the mean value of modulus of elasticity of the sample were determined according to EN 14358 [34]. Since there were more than 40 specimens, both parametric (P) and non-parametric (NP) approaches were used, and the results of characteristic values obtained from the two approaches were compared. EN 14358 [34] specifies that the parametric approach should not be used to test data that does not fit the assumed distribution and, therefore, a Kolmogorov-Smirnov goodness-of-fit test (K-S) was made to evaluate whether the population distributions assumed were correct (log-normal for bending strength, and normal for density and for modulus of elasticity). 

(iii) Population: The characteristic values of bending strength, modulus of elasticity, and density of the population were calculated for both P and NP approaches according to EN 384 [33].

### 2.4. CLT Manufacturing Process

**Figure 2:**
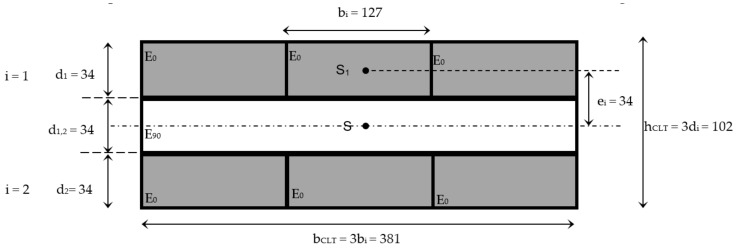
Layout scheme of cross-laminated timber (CLT) (dimensions in mm).

Twenty CLT panels were manufactured using sawn boards graded as CTH (see Section 2.1) of 3.00 m in length without finger joints, with an average MC of 13%. All boards were planed to a thickness of 34 ± 1.5 mm, just prior to gluing. Single-component polyurethane adhesive (PUR) is the most common adhesive used internationally for the manufacturing of CLT panels, although other adhesives have been identified as appropriate for the production of structural elements [18], such as phenolic and aminoplast adhesives (melamine urea formaldehyde -MUF-, melamine formaldehyde -MF-, urea formaldehyde -UF-, phenol resorcinol formaldehyde -PRF-, etc.) and emulsion polymer isocyanate adhesive (EPI). Since Uruguay is not a producer of CLT panels or of structural wood adhesives, but EPI adhesive (HEXION EPI 3S742) is commercially available in Uruguay, this is what was used in the manufacture of the panels. EPI was applied between the layers for 180 min at a spreading rate of 198 gr/m^2^ and a pressure of 10 N/mm^2^, following the recommendations of the manufacturer. During gluing, the temperatures varied between 15 and 22 °C and the relative humidity between 64 and 75%. Based on the nominal cross-section of the individual sawn boards (34 × 127 mm^2^), the nominal cross-section of the CLT panels was *b_CLT_* × *h_CLT_* = *b_i_* × 3*d_i_* = 381 × 102 = 38,862 mm^2^, as shown in Figure 2.

### 2.5. CLT Bending Tests

**Figure 3:**
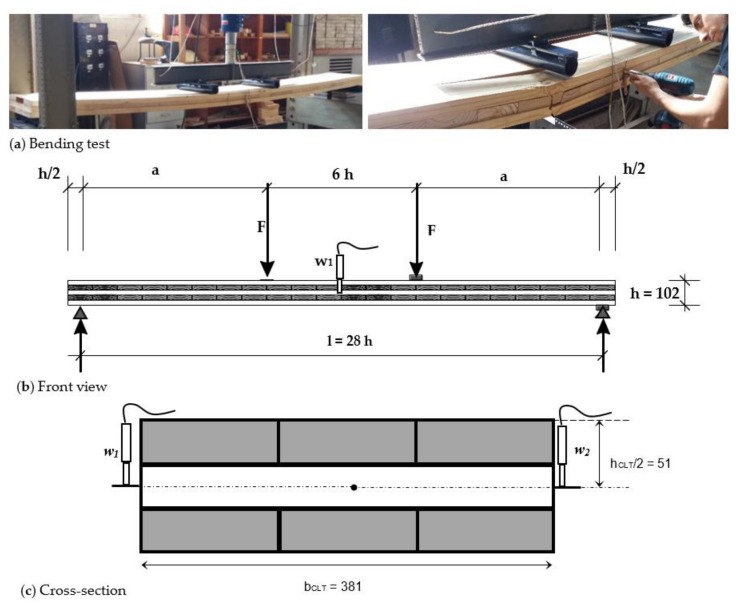
Configuration of the CLT bending test and the location of the linear variable differential transformers (LVDTs): (**a**) bending tests, (**b**) front view, and (**c**) cross-section (dimensions in mm).

CLT specimens were tested in bending with loads perpendicular to the plane according to the specifications of EN 16351 [17] (Figure 3). Since the ratio (*b_i_*/*t_i_*) was lower than 4, the test span considered was 28 times the CLT height in order to reduce the incidence of rolling shear failure. Global deflections were measured by LVDTs in the elastic phase of behavior at mid-span on both sides of the specimens. In addition, the maximum load at the moment of failure was registered.

### 2.6. Characterization of the CLT Panels

Although the bending test requirements of CLT panels are specified in EN 16351 [25], as mentioned previously, this standard does not provide a method to determine the corresponding modulus of elasticity and bending strength, unlike those for other engineered wood products (EWP) standards, such as GLT [26]. 

Longitudinal modulus of elasticity was calculated according to the Timoshenko beam theory (Equation (1)), with a value of net moment of inertia in accordance with Steiner’s Theorem (Equation (3)). It was assumed that all sawn boards, and therefore all layers, had the same longitudinal modulus of elasticity, and the modulus of elasticity perpendicular to the grain of the layers was taken as 0.

(3) $$I_{i\_ST}=\frac{b_{CLT\;}d_{i}^{3}}{12}+\;A_{i}e_{i}^{2}$$

where, *I_i_ST_* is the net moment of inertia of the layer *i* in relation to its neutral axis, calculated according to Steiner’s Theorem, which is valid for layers with the same modulus of elasticity; *A_i_* is the area of layer *i* (*A_i_* = *b_CLT_ d_i_*); and *e_i_* is the distance between the gravity center of the layer *i* (*S_i_*) and the gravity center of the CLT panel (*S*), Figure 2.

The bending strength of the CLT panels (*f_m,CLT_*) was calculated using Equation (4).

(4) $$f_{m\_CLT}=\;\frac{\frac{F_{max}}{2}a}{K_{CLT}}z\;E_{0\left(z\right)}$$

where, *F_max_* is the maximum load applied in bending at the moment of rupture; *a*, is the distance between the support and the point of load application; *z* is the distance between the neutral axis and the farthest point of the section (*z* = *e_i_* + *d_i_*/2 = *h*/2); *E_0_*_(*z*)_, is the modulus of elasticity of the layer in question; and *K_CLT_* is a measure of stiffness in bending, as defined by Equation (5).

(5) $$K_{CLT}=\;\sum \left(E_{0,i\;}I_{i\_ST}\right)$$

where, *i* is the longitudinal layer; *E*_0,*i*_ is the modulus of elasticity parallel to the grain of the layer *i*; and *I_i_ST_* is the net moment of inertia of layer *i* related to its neutral axis (Equation (3)).

The density of each layer *i* was defined as the average of the density of boards adjusted to the reference MC. The mean density of each CLT panel was taken as the lowest mean density of the layers [25].

The procedure for the determination of the characteristic values of bending properties and density followed the requirements of EN 14358 [34], as described in Section 2.3. A reference cross-section of 150 mm was proposed in the state-of-the-art report of the COST Action FP1402 [28] for tests in bending out-of-plane. Since the cross-section of the tested CLT panels had a cross-section of 102 mm, bending strength could be increased by the height factor (*k_h_*). However, the value of *k_h_* was conservatively assumed as 1.

### 2.7. Structural Performance

The sizing of CLT floors was analytically calculated in Ultimate Limit States and Serviceability Limit States (deflections and vibrations), using the Gamma method, following the recommendations published by proHolz [31], based on Annex B of Eurocode 5 [30], which use the characteristic values of the bending properties and density of Uruguayan pine graded as CTH (see Section 2.1). This method is based on the Bernoulli’s beam theory, and the shear deformations were not taken into account in the longitudinal layers. The Gamma method predicts the “effective stiffness” of CLT on the basis of the mechanical properties of the layers. It considers the longitudinal layers as elements which are connected with imaginary fasteners, represented by a “connection efficiency factor” (γ), which considers that using a measure of the effective stiffness of the panel is equivalent to considering the rolling shear deformation of the cross layers (G_R_). That is, a value of γ equal to 1 represents a completely glued longitudinal layer, and a value of 0 indicates no connection at all between the layers. Therefore, it seems reasonable to use the Gamma method for span to depth ratios over 30, as was the case in this study.

**Figure 4:**
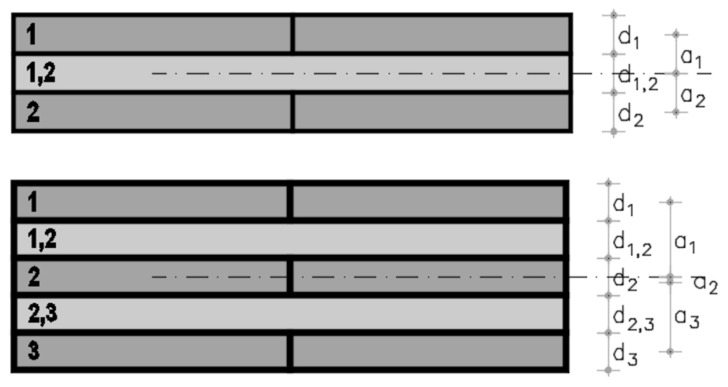
Layout of the 3- and 5-layer CLT according to the Gamma method.

Figure 4 shows the layout of the CLT panels to which the Gamma method was applied.

The bending stiffness for deformation (*K_CLT_*__γ_) was calculated according to the Gamma method, and the shear deformation was estimated by the effective moment of inertia (*I*_0,*ef*_). In the absence of experimental values for Uruguayan pine, the rolling shear modulus commonly used for softwood (G_R_ = 50 MPa) was assumed [18,31], which implies an increase in bending deformation because the shear deformations are being indirectly taken into account. This value agrees with the ratio G_R_/G = 0.1 defined in the Eurocode 5 [30] for softwood. A study on Scottish Sitka spruce found an increase of 4% in the modulus of elasticity when using the Gamma method compared to both the Timoshenko and Shear Analogy methods, probably due to the effect of the assumed value of rolling shear modulus [18]; so further research to determine the rolling shear stress and stiffness for Uruguayan pine is needed.

It should be noted that the distance *α_i_* (Figure 4) is not necessarily equal to *e_i_* (Figure 1). For 3-layer CLT, one of the external layers is taken as reference, and the symmetry of the section is thus lost, which implies that *α_i_* differs from *e_i_*. However, for 5-layer panels, taking the central layer as reference, the section remains symmetrical and *α_i_* is, therefore, equal to *e_i_*. 

The extended Gamma method was used for the sizing of 7-layer panels [31], where the γ values are determined by a linear equation system.

## 3. Results and Discussion

This section presents the experimental results of characterization of the sawn boards and the CLT panels, as well as the relationship between them, and the structural performance of CLT panels for flooring.

### 3.1. Mechanical Properties of Uruguayan Pine from Small-Diameter Logs

Since whorls of Uruguayan Loblolly/Slash pines are present approximately every 0.6 m, the probability of the presence of a knot in the central third of the tested beams is high. Furthermore, it is known that not only the knot diameter, but also the local grain deviation around knots influence ruptures, so that a combination of failure by tension parallel to the grain, tension perpendicular to the grain, and shear is reached in the vicinity of the knot [38]. Since the maximum diameter of knots for the sawn timber graded as CTH is 4/5 h (see Section 2.1), probability of the presence of a knot in the tensile side of the beams is also high. In agreement with this, results showed that 73% of the tested beams broke by the influence of a knot (51% in the central third and 22% outside of the central third) and 27% by tension parallel to the grain without influence of knots. 

The Kolmogorov-Smirnov goodness-of-fit test (K-S) proved that the population distribution assumption was correct (see Section 2.3): significance levels of 0.79, 0.84, and 0.97 for bending strength, modulus of elasticity, and density, respectively. Table 1 shows the mean and the characteristic values of the longitudinal modulus of elasticity, bending strength, and density for Uruguayan Loblolly/Slash pine from thinning graded as “CTH” (see Section 2.1), from both P and NP approaches. 

It can be observed that the characteristic values of bending strength and density with the NP approach were, respectively, 9.3% and 2.4% higher than those obtained using the P approach for a single sample of 45 specimens. Consequently, in order to verge on the side of caution, the calculations/estimations of the structural properties of the sawn timber boards comprising the layers used in the CLT structural design (see 5.3) that were used in subsequent calculations were those obtained from the P approach: *f_m,k_P_* = 6.9 MPa; *E_0,mean,k_* = 6805 MPa; and *ρ_k_P_* = 326 kg/m^3^.

In a comparison between the experimental bending properties of the Uruguayan pine graded as “CTH” and the requirements of the strength class C14 (the lowest strength class defined in EN 338 [7]), the experimental modulus of elasticity was slightly (2.8%) below that required by the guidelines. However, bending strength was 51% below that specified for grade EC7 (C14 strength class), due to the knot size permitted by the grade CTH being double that acceptable for grade EC7. 

### 3.2. Relationship between the Structural Properties of the Layers and Those of the CLT Panels

**Figure 5:**
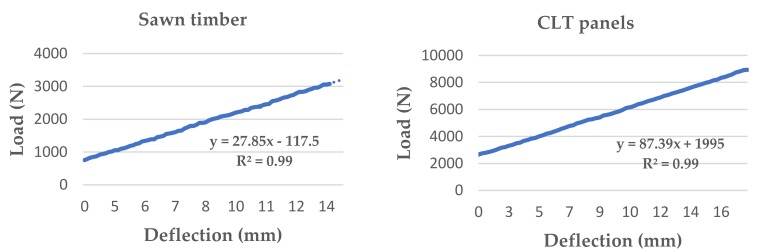
Load-displacement relation for a sawn timber beam and a CLT panel.

A comparison was made between the bending properties of the CLT panels obtained with the two different approaches defined in EN 16351 [25] (i.e., the determination of the properties of the individual layers and testing of the CLT panels themselves). Load-displacement relations for one sawn timber beam and one CLT panel are shown in Figure 5. The characteristic values of bending strength, longitudinal modulus of elasticity, and density were estimated according to the P approach [13] for the samples, the results of which are shown in Table 2. EN 16351 [25] does not include equations to adjust the characteristic values of the sample to the population, therefore, the characteristic values of the population were estimated according to EN 384 [33]. 

**Figure 6:**
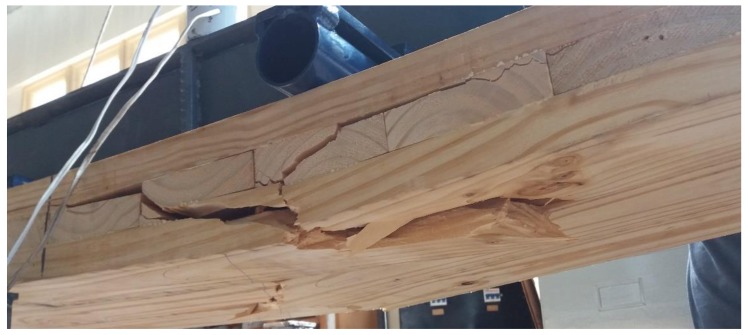
Tensile failure on the bottom of the CLT followed by rolling shear failure.

Most of the CLT panels tested (85%) showed a tensile failure initiated by (i) the influence of a knot in the lower layer (60%), which was commonly followed by rolling shear failure in the cross layer (Figure 6); and (ii) a combined failure between tension in the lower longitudinal layer and shear in the cross layer (25%). The observed failure behavior was similar to what is described for black spruce [39]. The orientation of the annual ring of the cross layers of the CLT panels tested was aleatory. Since the orientation of the annual rings in the cross-section influences the shear strain distribution and the rolling shear modulus [40,41], future works could focus on the study of the influence of annual ring orientation and the presence of pith in shear tensions of the cross layers. The remaining 15% showed a failure due to delamination on the glue line, which suggests the need for future research into the strength of the bond between lamellas before entering into commercial production of CLT panels made from small logs of Uruguayan Loblolly/Slash pine. 

The standard for glued laminated timber (GLT), EN 14080 [26], relates the strength class of the GLT beams with that of the sawn boards used as raw material. Mechanical properties of GLT are usually equal or higher than that of the raw material when the manufacturing process is in accordance with the minimum requirements described in the standards; e.g., GLT of strength class GL20h (bending strength, *f_m,k_* = 20 MPa) can be composed by sawn boards of strength class C16 (*f_m,k_* = 16 MPa) [18]. With the idea of proposing strength classes for CLT panels manufactured using sawn boards graded as CTH, characteristic values of the bending properties of the CLT panels were compared with that of sawn boards (Table 1). 

The results show that the bending strength of the CLT panels was 43% higher than that of the individual sawn timber boards in the layers, which were graded as CTH, while the mean value of the local modulus of elasticity was 13% lower. Furthermore, it was observed that the local longitudinal modulus of elasticity of the CLT panels was 3% higher than their global modulus of elasticity (*E_g_* = 6198 MPa), which was calculated assuming a value of G equal to ∞. 

The results obtained in this work are in accordance with the behavior observed for hybrid poplar [42], where the bending strength of CLT panels was found to be slightly increased with respect to the mechanical properties of the layers themselves, and with lower values for the modulus of elasticity. Similarly, the modulus of elasticity of CLT panels of Irish Sitka spruce was found to be between 5 and 18% lower than that of the layers comprising the panels (strength class C16), while the bending strength of panels was between 17 and 36% higher than that for layers [18]. 

The results reported in the present paper were obtained for panels manufactured with boards without finger joints. Although finger joints influence the bending strength, this influence is likely to be negligible in boards with many defects, such as knots. For example, Lara-Bocanegra et al. [43] showed that the bending strength of boards of *Eucalyptus globulus* with finger joints was 23% lower than that of boards made of “clear wood”. However, the characteristic values of the bending strength of the finger joint itself were up to 37% higher than that required for strength class D40 for sawn boards of this species. Similar results were found by Piter et al. [44] for *Eucalyptus grandis*, where bending strength of the finger joint was 76% higher than that for the declared strength class of the board.

It is also important to note that the number of specimens and samples used influences the characteristic values, and it is thus important to identify whether the values reported in studies refer to the sample or to the population. The results of the present work showed that the population characteristic values were 30% lower than the sample values for bending strength and 7% lower for modulus of elasticity. In turn, the characteristic value of panel density was 388 Kg/m^3^ for the sample and 341 Kg/m^3^ for the population, both very similar to the density of the layers themselves.

### 3.3. Effect of Strength Class on the Structural Performance of CLT Floors

The objective of this study was to evaluate the structural performance of CLT floors made from Uruguayan pine in relation to the most common commercial CLT panels sold internationally, which are manufactured with longitudinal boards of strength class C24. The bending calculations of the panels were made with the Gamma method, using the mechanical properties of the layers, for the imposed loads for buildings according to the following categories of use [45]: A-residential areas (2 kN/m^2^); B-office areas (3 kN/m^2^); C-public concurrence areas (4 kN/m^2^); and D-shopping areas (5 kN/m^2^). 

A theoretical analysis of the structural yield in bending was made according to the number of layers (3 to 7) with thicknesses varying between 18 to 60 mm, and the effect of strength class (C14, “CTH” or C24) of the layers on the maximum span was studied.

**Figure 7:**
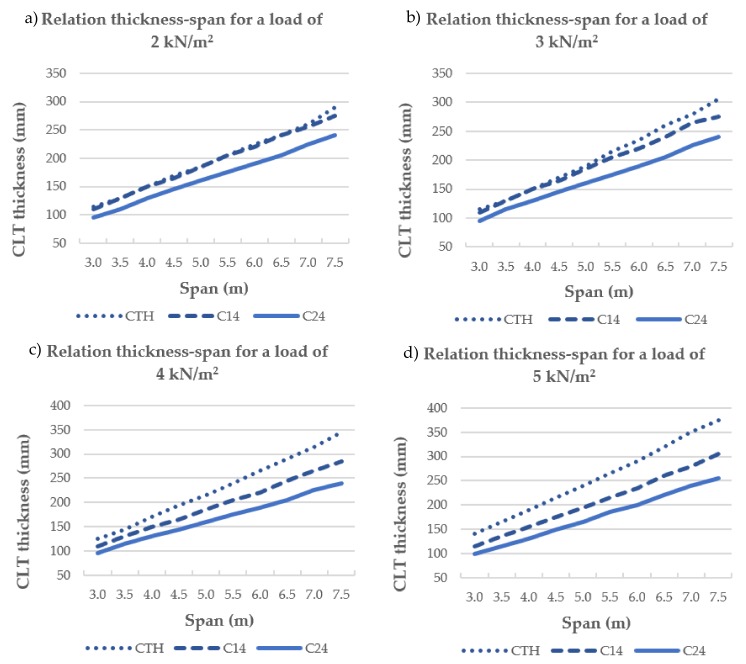
Relation thickness–span in the 5-layer CLT for different strength classes: (**a**) imposed load = 2 kN/m^2^, (**b**) imposed load = 3 kN/m^2^, (**c**) imposed load = 4 kN/m^2^, and (**d**) imposed load = 5 kN/m^2^.

Figure 7 shows the thickness-span relationship in 5-layer CLT depending on strength class and load. 

The thickness–span relation for loads below 4 kN/m^2^ was very similar for panels made from CTH (spotted line in Figure 7) or C14 (stripped line in Figure 7) grade-layer because the design is governed by the modulus of elasticity in Serviceability Limit States (SLS) and more specifically by the verification of deflection due to a unit force of 1 kN. However, since the modulus of elasticity of CTH (6805 MPa) and C24 (11000 MPa) differed from one to another, the difference between the thickness-span relation for CTH and C24 (solid line in Figure 7) panels varied between 17 and 21% (Figure 7a,b). For these loads, the use of pine graded as CTH implies an increase of total thickness of CLT floors of between 14 and 26% with respect to C24, depending on the number of layers. 

Higher relative differences in the thickness–span relationship were observed for higher loads (Figure 6c,d) where the design is governed by strength in Ultimate Limit States for CTH timber, and by modulus of elasticity in SLS (deflection and vibration) for C14 and C24. The maximum difference was 31% between CTH and C24 for an imposed load of 5 kN/m^2^ (Figure 6d).

A similar trend was observed for 3- and 7-layer CLT, and the relative differences of those relationships for the three strength classes were calculated and they are shown in Table 2.

The analysis of the maximum span for 3-, 5-, and 7-layer CLT, with a constant layer thickness of 33 mm, for the different strength classes and categories of use, is shown in Table 3. The property and/or the limit state are identified by the following superscripts: (D) when the deflection was the limit value; (V) when the sizing was defined by vibration; (V_d_) when the sizing was defined by vibration due to a static load of 1 kN; and (B) when the failure was reached by bending strength in the ultimate limit state (ULS).

Serviceability Limit States (D, V, and V_d_) governed the sizing of the CLT panels when they were manufactured using timber of strength classes C14 or C24. However, bending strength became the limiting factor in the sizing of panels made with CTH timber for high imposed loads. 

There was no difference in the maximum span (3.0 m) for either the CTH grade or the C14 strength class for 3-layer CLT loaded up to 4 kN/m^2^. This was due to the fact that the design was governed by the modulus of elasticity in SLS verifications, the values of which were very similar for both grades. 

The maximum span for 5-layer CLT made with pine graded as CTH, and for loads up to 4.0 kN/m^2^, was between 10 and 20% shorter than when using timber of strength class C14. This is because, while panels made with layers of strength class C14 are sized by SLS, panels made with layers graded as CTH are limited by ULS. Similar behavior was observed for 7-layer panels, with spans for CTH panels being 18% lower than those of C14. 

In an analysis of the volume of wood used in the construction of a building, the use of CTH timber implies an average wood volume per building area (m^3^/m^2^) 17% higher than that using C24 timber. However, this volume increase could be counterbalanced by the cost of the panels, especially if logistic costs are considered. 

## 4. Conclusions

A new visual grade, named CTH, was established for Uruguayan Loblolly/Slash pine from thinning operations, whose bending strength was 51% lower than that corresponding to the minimum strength class (C14) as defined in the European Standard EN 338 [13]. CLT panels were made using this low-strength timber, and experimental results showed that the bending strength of the resulting panels increased by 43% with respect to the sawn timber, while the modulus of elasticity was 13% lower.

The influence of the method used to calculate the characteristic values of the structural properties is important, and, for a single sample, characteristic values obtained from non-parametric approaches are higher than those obtained from parametric approaches. Likewise, it is important to identify whether the characteristic values reported in trials correspond to the sample or to the population. Results of the present work showed a decrease in characteristic values of the population of between 30% and 7% with respect to those of the sample. 

This work demonstrates the structural viability of using Uruguayan Loblolly/Slash pine of low mechanical properties to design CLT. Furthermore, the timber volume of Uruguayan CLT with respect to the most common commercial strength class (C24) CLT was quantified, obtaining a 14%- and 26%-increase in the total thickness for 3-layer CLT and 7-layer CLT, respectively, according to guidelines for residential and office buildings. 

The analysis of the influence of the strength class on maximum span showed that the use of Uruguayan pine implies a decrease of between the 9 and 29% in maximum span with respect to C24. In an analysis of the average timber volume per building area (m^3^/m^2^), 17% more wood is necessary if CTH strength class is used instead of C24.

Future research is needed to evaluate the economic feasibility of fabricating Uruguayan CLT panels, mainly associated with the rolling shear strength and stiffness in the cross layer, shear strength in the glue line, and the influence of the low density of the wood on the performance of panels in compression perpendicular to the grain and in connections. Further estimation of the market price of Uruguayan CLT is required to establish whether the increase in timber volume is compensated for by its lower price. Furthermore, fracture mechanics tests should be analyzed to understand the tenacity of Uruguayan CLT.

## Figures and Tables

**Table 1:** Bending properties and density of the CTH sawn timber and the CLT panels. CTH is the newly proposed visual grade.

Sample					CTH Sawn Timber	CLT Panels
Number of specimens			n		45	20
Cross-section			Height (h)	mm	127	102
			Width (b)	mm	37	381
Moisture content (MC)			MC mean	%	13.4	13.0
			COV	%	7	6
Bending Strength ^a^	Mean Values		*f_m_mean_*	MPa	19.8	21.2
			COV	%	32	20
	Characteristic Values	Sample	*f_mk,s_P_*	MPa	9.9	14.2
			*f_mk,s_NP_*	MPa	10.8	-
		Population	*f_mk_P_*	MPa	6.9	9.9
			*f_mk_NP_*	MPa	7.6	-
Modulus of Elasticity ^b^	Mean Values Sample		*Ē__12_*	MPa	7347	6383
			COV	%	29	23
	Characteristic Values Population		*E_0,mean,k_*	MPa	6805	5913
Density ^b^	Mean Values		*ρ__12,mean_*	Kg/m^3^	440	429
			COV	%	9	5
	Characteristic Values	Sample	*ρ_k,s_P_*	Kg/m^3^	370	388
			*ρ_k,s_NP_*	Kg/m^3^	380	-
		Population	*ρ_k_P_*	Kg/m^3^	326	341
			*ρ_k_NP_*	Kg/m^3^	334	-

NP and P stand for non-parametric and parametric approach, respectively, and COV the coefficient of variation. ^a^ Adjusted to a reference depth of 150 mm for CTH sawn timber; ^b^ Adjusted to a reference moisture content of 12% for both sawn timber and CLT panels.

**Table 2:** Relative difference of the relationship thickness–span of the CTH with respect to C14 and C24.

		δ (%) 3-Layer CLT				δ (%) 5-Layer CLT				δ (%) 7-Layer CLT			
	Load	2 kN/m^2^	3 kN/m^2^	4 kN/m^2^	5 kN/m^2^	2 kN/m^2^	3 kN/m^2^	4 kN/m^2^	5 kN/m^2^	2 kN/m^2^	3 kN/m^2^	4 kN/m^2^	5 kN/m^2^
SC													
CTH-C14		0–2	0–4	5–12	15–16	4–5	4–10	12–17	18–19	0–7	0–13	11–17	19–21
CTH-C24		14–16	14–18	19–26	27–29	17–17	17–21	24–30	29–32	16–18	18–26	26–31	29–33

δ is the relative difference and SC is the strength class.

**Table 3:** Maximum span (m) for the different strength classes, loads, and number of layers.

Imposed Loads (kN/m^2^)	7-Layer CLT			5-Layer CLT			3-Layer CLT		
	CTH	C14	C24	CTH	C14	C24	CTH	C14	C24
**2**	6.5 ^D/V/B^	6.5 ^V_d_^	7.5 ^V^	4.5 ^V_d_^	5.0 ^V_d_^	5.5 ^V_d_^	3.0 ^V_d_^	3.0 ^V_d_^	3.5 ^V_d_^
**3**	6.0 ^B^	6.5 ^V_d_^	7.5 ^V^	4.5 ^D/V/B^	5.0 ^V_d_^	5.5 ^V_d_^	3.0 ^V_d_^	3.0 ^V_d_^	3.5 ^V_d_^
**4**	5.5 ^B^	6.5 ^D/V^	7.5 ^D/V^	4.0 ^B^	5.0 ^D/V^	5.5 ^D/V^	3.0 ^D/B/V^	3.0 ^V_d_^	3.5 ^V_d_^
**5**	5.0 ^B^	6.0 ^D^	7.0 ^D^	4.0 ^B^	4.5 ^D^	5.5 ^D^	2.5 ^B^	3.0 ^D/V^	3.5 ^D/V^

D is deflection; V is vibration; V_d_ is vibration due to a static load of 1 kN; and B is bending strength.
